# A Chitosan–Agarose Polysaccharide-Based Hydrogel for Biomimetic Remineralization of Dental Enamel

**DOI:** 10.3390/biom11081137

**Published:** 2021-08-02

**Authors:** Viorica Muşat, Elena Maria Anghel, Agripina Zaharia, Irina Atkinson, Oana Cătălina Mocioiu, Mariana Buşilă, Petrică Alexandru

**Affiliations:** 1 Laboratory of Nanochemistry, Centre of Nanostructures and Functional Materials-CNMF, “Dunărea de Jos” University of Galaţi, 800201 Galaţi, Romania; 2 Institute of Physical Chemistry “Ilie Murgulescu” of Romanian Academy, 060021 Bucharest, Romania; irinaatkinson@yahoo.com (I.A.); omocioiu@icf.ro (O.C.M.); 3 Faculty of Dentistry, Ovidius University of Constanta, 900573 Constanţa, Romania; agrizaharia@yahoo.com; 4 Department of Materials and Environmental Engineering, Centre of Nanostructures and Functional Materials-CNMF, “Dunărea de Jos” University of Galaţi, 800201 Galaţi, Romania; mariana.ibanescu@ugal.ro (M.B.); palex@ugal.ro (P.A.)

**Keywords:** natural polysaccharides-based hydrogel, acid-etched native enamel, agarose, chitosan, biomimetic remineralization, hydroxyapatite

## Abstract

Developing multifunctional systems for the biomimetic remineralization of human enamel is a challenging task, since hydroxyapatite (HAP) rod structures of tooth enamel are difficult to replicate artificially. The paper presents the first report on the simultaneous use of chitosan (CS) and agarose (A) in a biopolymer-based hydrogel for the biomimetic remineralization of an acid-etched native enamel surface during 4–10-day immersion in artificial saliva with or without (control group) fluoride. Scanning electron microscopy coupled with energy-dispersive X-ray spectrometry, Fourier transform infrared and Raman spectroscopies, X-ray diffraction, and microhardness tests were applied to investigate the properties of the acid-etched and remineralized dental enamel layers under A and CS-A hydrogels. The results show that all biomimetic epitaxial reconstructed layers consist mostly of a similar hierarchical HAP structure to the native enamel from nano- to microscale. An analogous Ca/P ratio (1.64) to natural tooth enamel and microhardness recovery of 77.4% of the enamel-like layer are obtained by a 7-day remineralization process in artificial saliva under CS-A hydrogels. The CS component reduced carbonation and moderated the formation of HAP nanorods in addition to providing an extracellular matrix to support growing enamel-like structures. Such activity lacked in samples exposed to A-hydrogel only. These data suggest the potential of the CS-A hydrogel in guiding the formation of hard tissues as dental enamel.

## 1. Introduction

The deterioration of human dental enamel can greatly influence both your attitude on life, because of how you look and feel, as well as your health. As a non-living tissue, much to our regret, dental enamel does not easily regenerate and remineralize. Dental enamel consists of mostly c-orientated carbonate hydroxyapatite nanorods (prisms). These are organized in bundles whose boundaries are delimitated by interprismatic crystallite and organic covering [1,2]. The unique anisotropic mechanical behavioral properties of the dental enamel [3,4], which result in a distinct organic pattern, control the self-organization of the inorganic component (HAP) into the complex hierarchical structure.

Traditional treatments of damaged enamel imply removing carious affected areas and using restorative materials, such as resin composites and ceramics. Patients who suffer from numerous decay treatments may undergo permanent loss of the tooth structure. How long a dental restoration will last can depend on the seriousness of the original cavity problem, the patient habits, the material used to rectify the tooth, as well as dentist abilities [5,6,7,8].

The search for a non-invasive treatment to prevent cavities has led to a use of biochemical methods for tooth remineralization [9,10]. The biochemical method of extracellularly-mediated biomineralization of human enamel takes place during tooth development. In this way, enamel mineralization is assisted by an organic matrix with 90% amelogenin, which is an enamel protein secreted by ameloblasts during the first stage of the enamel formation (amelogenesis) [1,3]. By amelogenesis completion, these biomolecules degrade and/or are removed gradually. Therefore, the main role of the organic matrix consists of creating templates for both nucleation control and the growth modulation of the HAP crystals.

Enamel reconstruction by the biomimetic remineralization method imitates natural mineralization processes and is a novel approach for treatment of the early-stage eroded enamel, white spots, and/or non-cavitated lesion as well as for the prevention of cavity progression [3,10,11]. The biomimetic process of enamel remineralization is guided by an organic self-assembling matrix that develops scaffolds where HAP crystals nucleate and grow from a remineralizing environment, yielding enamel-like structures. The most common remineralizing media for restoring the functionalities of the dental enamel are artificial and natural saliva, simulated body fluids, slurries of bioglasses, and hydroxyapatite [11]. The biochemical methods of enamel repair are cell and/or protein-matrix-assisted methods [12,13]. Although the formation of human dental enamel is a result of cellular and chemical processes [1], the cell-assisted (xenogenic and stem cells) hard tissue remineralization is rather complicated [1,12], so that cell-free solutions are preferred. Protein [14,15] and protein analogues [16,17,18,19] were used to obtain extracellular environments guiding the construction of enamel-like apatite layers on the surface of enamel lesions [1,12]. However, natural proteins such as commercially available EmdogainR (EMD) extracted from porcine teeth [18] are difficult to obtain. Peptides and oligopeptides are known as protein analogues providing scaffolds for HAP nucleation and promoting the remineralization of dental enamel [9,11,16,17]. A combination of peptides and fluorides is a new biomimetic way reported as a treatment for the carious lesions of enamel [19].

Some reports [20,21] indicate that agarose provided a good biomimetic microenvironment for human enamel and dentine remineralization while repairing the loss of non-carious tooth enamel when covered by ionic and non-ionic agarose hydrogel. Agarose (A), a member of the natural polysaccharide family extracted from red algae, has determined the formation of thermo-reversible hydrogels mimicking gel-like organic matrices for growing of the prism-shaped HAP crystals [21]. Due to its repetitive anionic -OH groups, agarose is among the non-collagenous biological materials [13] used as a template in tissue engineering to control the self-assembly of the HAP nanocrystals into nanorod crystals [20]. Along with excellent biocompatibility and low cost, the agarose tunable properties make it suitable for various medical applications such as regeneration of cartilage [22], bone [23,24] as well as drug delivery systems [21]. Han et al. [25] used an agarose hydrogel loaded with Ca^2+^ and PO_4_^3−^ ions for rabbit dentine’s remineralization. Even if it was confirmed that agarose hydrogel has a high potential into human enamel remineralization [20], more dedicated studies are necessary to clarify the biomimetic effect and stability of agarose hydrogels in clinical dental application.

Another potential candidate for biomimetic enamel matrix is chitosan. Chitosan (CS), a well-known linear polysaccharide exhibiting low cost, biodegradability, biocompatibility, and anti-inflammatory properties [26,27], can form hydrogels by its dilution in acetic acid with preventive and therapeutic roles for dental caries [26]. The biocompatible, biodegradable, and non-toxic natural polysaccharide biopolymers not only provide a substrate to immobilize the HAP nano-building units but also can act as a template for their structural assembly [27,28]. Many papers reported the CS effect as a releasing agent in the remineralization mechanism under amelogenin–chitosan hydrogel matrix to stabilize Ca-P clusters and promote their assembling into organized crystals [29,30,31]. In contrast with agarose, stable hydrogel networks containing chitosan are obtained by chemical crosslinking. Recently, Garakani et al. [32] successfully obtained nasal cartilage scaffolds under a crosslinked chitosan–agarose system. In addition, bone scaffolds were prepared from a paste containing chitosan, agarose, and nanohydroxyapatite [33]. Dextrin crosslinked chitosan–agarose-based hydrogels with tuned physical and biomechanical characteristics were developed for multifunctional soft tissues regeneration [34], but there is no report for their use in enamel regeneration. The biomimetic growth of acid-etched coronal human dentine under chitosan–agarose hydrogels action was reported in the literature [35]. In the latter case, non-toxic and difficult to remove crosslinking agents such as glutaraldehyde and potassium persulfate [36,37] were used for chitosan. Although biomimetic methods can treat only tooth microdefects at the current stage, the potential for tooth defect repair via a self-healing mechanism would be ideal to use in dental clinics [10,11,16].

The purpose of this work is to induce the biomimetic remineralization of acid-etched human enamel in artificial saliva containing fluoride under agarose and novel chitosan–agarose hydrogels action without a toxic crosslinking agent for chitosan. Chitosan addition to the agarose hydrogel was seen to induce a biomimetic effect closer to that of the proteins in the organic matrix during formation of the enamel. Hence, combined agarose–chitosan and fluoride effects on biomimetic enamel remineralization were studied. The morphology, chemical composition, crystalline structure, and microhardness of the new remineralized layers grown for four to ten days onto the acid-etched enamel surface were investigated by scanning electron microscopy coupled with energy-dispersive X-ray spectrometry, X-ray diffraction (XRD), Fourier transformed infrared (FTIR), and micro-Raman spectroscopies, as well as Vickers microhardness measurements.

## 2. Materials and Methods

### 2.1. Tooth Slice Preparation

Selection and tooth slice preparation of the caries-free human molars were carried out in accordance with patients consent and ethical protocols of the Ovidius University of Constanta, Faculty of Dentistry under registration number (169–01-17). Ten caries-free molars were disinfected by treatment with 3 wt. % sodium hypochlorite (NaClO) [38] and phosphate-buffered saline (PBS). The cleaned molars were longitudinal sectioned with a low-speed diamond disk under air-cooling. Four slices (1.5–2 mm thick) with an outer face of the tooth crown of native enamel were selected for each tooth. These slices were two buccolingually, and the other two were mesiodistally. The demineralization of slices carried out in Blue Etch acid for 60 s was followed by three times rinsing for 2 min with deionized water in an ultrasound bath. Next, the tooth slices were dried and stored at 4 °C prior to remineralization treatment.

### 2.2. Preparation of Remineralized Coatings

The remineralization treatment of the damaged enamel was performed under biomimetic hydrogel-based double-diffusion systems (DDSs) [39], namely CS-A polysaccharide hydrogels and artificial saliva (AS) with and without sodium fluoride addition.

Powders of chitosan (deacetylation degree of 75–85% and medium molecular weight of 190–310 KDa) and agarose (CSL-AG100) from Sigma-Aldrich (St. Luis, MO, USA) and Cleaver Scientific Ltd. (Rugby, UK) were used as received unless otherwise noted. Two types of agarose-based hydrogels, without (hydrogel-1) and with (hydrogel-2) CS, are presented in this paper. In short, the mixing of 0.95 g CaCl_2_ with 0.25 g agarose (A) into 50 mL of deionized water was carried out at 25 °C (room temperature, RT) under magnetic stirring. for complete dissolution. Then, this solution was placed in a closed vessel (ground stopper) and kept for 30 min. at 60 °C in an oven. Then, 150 μL of CaCl_2_ containing agarose solution and 15 μL of deionized water were added and mixed with 960 μL acetic acid solution with chitosan. The latter solution was prepared by adding 1% (*w*/*v*) chitosan in a 1% (*v*/*v*) acetic acid solution at RT under stirring at 80 °C until complete dissolution. The obtained hydrogel (A) had a pH of 6.5. After cooling at room temperature, the obtained solution was filtered by means of a 0.8 μm filter. The CS-A hydrogel was obtained by mixing 150 μL of CaCl_2_ containing agarose solution (prepared as mentioned before) and 15 μL of deionized water with 960 μL 1% chitosan solution (1% (*w*/*v*) chitosan in a 1% (*v*/*v*) acetic acid solution) with pH adjustment to 6.5. Artificial saliva (AS) was prepared according to Fletcher’s recipe, using 0.2 mM MgCl_2_, 1 mM CaCl_2_, 4 mM Na_2_HPO_4_, 16 mM KCl, 4.5 mM NH_4_Cl, and 20 mM HEPES (4-(2-hydroxyethyl)piperazine-1-ethane-sulfonic acid) buffer, adjusting the pH to 7.0 and adding sodium fluoride (300 ppm) before using [40]. For remineralization treatment, two series of cleaned dental slices were covered with 1M CaCl_2_ solution for 15 min followed by the application of a second layer of A or CS-A hydrogel, which was left to act for 2 h in a dryer at 37 °C. The dried slices were transferred into a vial with 30 mL of artificial saliva (AS) for biomimetic growing of the enamel-like structures at 37 °C during 4, 7, and 10 days, respectively. Tooth slices treated with CS-A hydrogel, further soaked in fluoride containing artificial saliva for 4, 7, and 10 days were noted CS-A-(4/7/10), while the ones without chitosan were named A-(4/7/10). The control groups without fluoride treatment during 4 and 7-day incubation were denominated CS-A-0F-(4/7) and A-0F-(4/7), respectively. At the end of the immersion period, tooth slices were removed, rinsed lightly with distilled water, and air-dried.

### 2.3. Morphological and Compositional Characterization

Scanning electron microscopy coupled with energy-dispersive X-ray spectroscopy technique, SEM-EDX, by FEI Q 200 microscope (FEI Czech Republic S.r.o., Brno, Czech Republic) in low vacuum conditions (0.45 Torr, spot sizes 2, 3, and 4, accelerating voltage of 10 and 20 kV, respectively), were used to investigate the morphology and elemental compositions of the remineralized coatings. To increase the sample conductivity, a 4 nm thick Au layer was deposited on the top of each examined sample, using an SPI-Module™ sputter coater system (SPI Supplies Division of Structure Probe, Inc., West Chester, PA, USA).

### 2.4. Structural Analysis

XRD measurements were performed using an Ultima IV- type Rigaku diffractometer (Rigaku corporation, Tokyo, Japan) equipped with parallel beam optics and a thin film attachment using Cu radiation (λ = 1.54056 Å) operating at 40 kV and 30 mA. XRD patterns of the studied slices were recorded over the 2θ range 10–80°, with a step size of 0.02 and a scanning rate of 5°/min. The fixed incidence angle, α, was set at 0.3° to determine the surface structure. Rigaku’s PDXL software version 1.8 was used for phase identification and crystallite size calculation. Crystallite size has been calculated using Scherrer’s equation along the (002) direction [41]. The crystallinity degree, *χ_c_*, representing the fraction of crystalline phase present in the sample has been calculated with the relation:

$$\chi_{c}={\left(\frac{K}{\beta \left(002\right)}\right)}^{3}$$

where *K* is a constant (for a great number of hydroxyapatites, *K* is 0.24), and *β* (002) is the peak (002) full width at half minimum (in degrees) [42].

Raman spectra of the remineralized dental slices and references were recorded by using a LabRam HR800 spectrometer (HORIBA FRANCE SAS, Palaiseau, France) equipped with an Ar^+^ excitation laser. An additional UV laser line (325 nm from Kimmon Koha Co LTD, Tokyo, Japan) was used to avoid highly fluorescent signals and to obtain surface Raman information. Two objectives of 100×/NA 0.9 and 40×NUV/0.47 of an Olympus microscope ensure a laser spot on the tooth surface up to 0.69 μm and 0.8 μm, respectively. The laser power was kept below 8 mW (514 nm) and 2.5 mW (325 nm) to avoid heating and sample degradation. Tooth slices were mounted on a computer-controlled, high-precision x-y stage with a spatial resolution of 0.05 mm. Spectra were background corrected and fitted by means of the PeakFit 4.12 software in order to obtain accurate peak parameters (position, full width at half maximum, FWHM, amplitude, and area). Fourier transform infrared (FTIR) spectra of the investigated samples were recorded in transmission mode without additional slice preparations, in the 400–4000 cm^−1^ domain with a spectral resolution of 4 cm^−1^, using a Thermo Nicolet 6700 spectrometer (Thermo Scientific Corporate, Waltham, MA, USA).

### 2.5. Mechanical Characterization 

The mechanical behavior on the cross-section of the new biomimetically remineralized and grown layers (biomimetically grown layers) in the presence of A and CS-A hydrogels was evaluated by Vickers microhardness measurements with 0.981 N (100 gf) loading for 15 s, using the PMT-3 type equipment (LOMO, St. Petersburg, Russia) with a digital camera (L3CMOS14000KPA, 14MP, ToupView software, Hangzhou, China). Five tests for every three different microzones on the analyzed surface were performed for each sample. Microhardness calculations were carried out according to standard test method ASTM E384-17.

## 3. Results

### 3.1. Morphologic and Structural Characterization of Biomimetic Remineralized Enamel 

**Figure 1:**
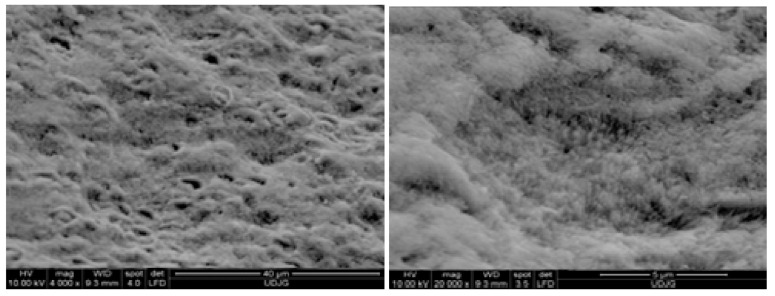
SEM top view images of acid-etched enamel reference sample (R-02).

**Figure 2:**
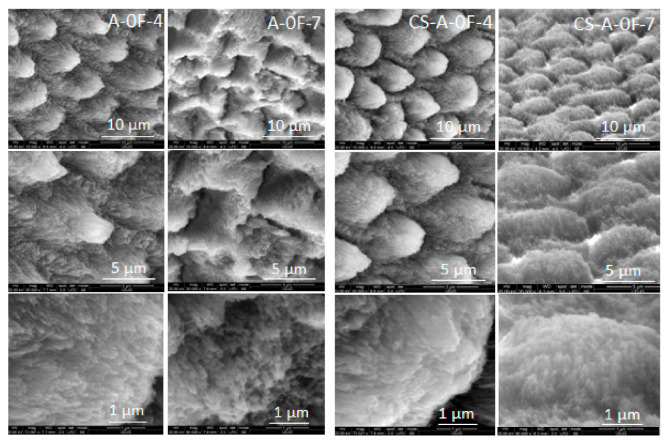
Top-view SEM images of the remineralized A-0F-(4/7) and CS-A-0F-(4/7) samples.

**Figure 3:**
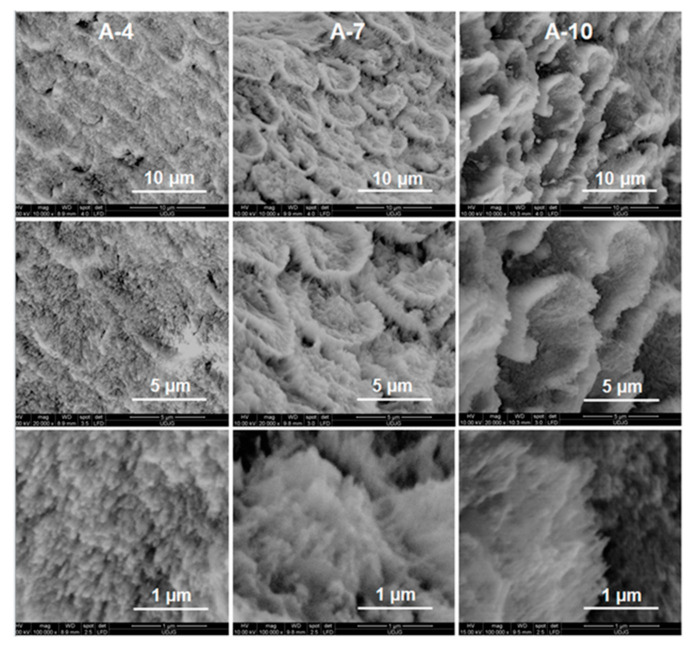
Top-view SEM images of the remineralized acid-etched enamel samples under agarose-based hydrogel for four (A-4), seven (A-7), and ten (A-10) days.

**Figure 4:**
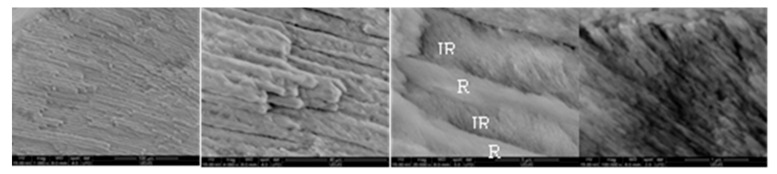
Longitudinal-section SEM images of the A-4 sample (IR and R is interrod and rod, respectively).

The top-view SEM images of the acid-etched enamel reference sample (R-02) in Figure 1 show integrity loss with irregular rods top end structure and high porosity [43,44,45]. Conversely, micrographs of all remineralized samples (Figure 2, Figure 3 and Figure 4) exhibit diminished microporosity. The two series of samples with and without chitosan, A-0F and CS-A-0F (Figure 2) grown in the absence of fluoride show an acicular morphology of the newly formed HAP crystals. More accentuated growth in the central rod area is depictable for both 4-day incubated A-0F-4 and CS-A-0F-4 samples. There are major differences between the morphology of the newly formed layers for seven days in accordance with the absence/presence of CS. Thus, SEM images of the A-0F-7 sample indicate an important increase in the interrod areas, forming a porous structure (Figure 2). Elsewhere, a comparable growth is observed in both rod and interrod areas of the CS-A-0F-7 samples, which are slightly accentuated in the central rod area, with compact appearance (Figure 2). However, the chitosan effect seems to reduce the diameter of the nanorods, from 30–60 nm (A-0F) to 20–40 nm (CS-A-0F).

For remineralized layers of the A-(4/7) samples (Figure 3), the large-scale distribution of the HAP rods–interrods embedded into sheaths was preserved, forming in cross-sectional view the keyhole-like structures of ≈5–5.5 μm in diameter. The keyhole-like structures run perpendicularly on the dentine–enamel interface up to the outmost surface of the dental enamel [45]. Thus, the axial section SEM micrographs of A-4 (Figure 4) show parallel bundles of HAP growth crystal rods and the corresponding assembled structures, which are identical to those from the native enamel.

**Figure 5:**
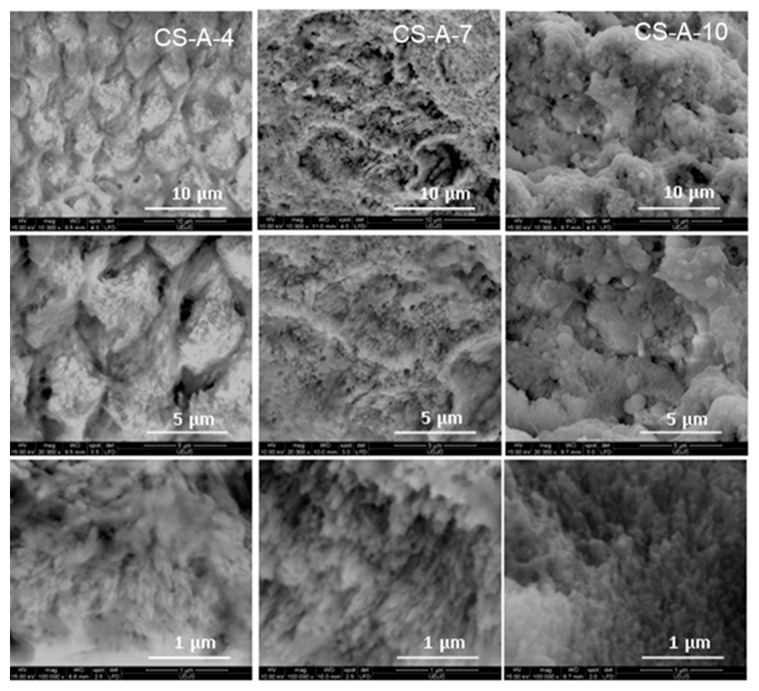
Top-view SEM images of the remineralized acid-etched enamel samples under chitosan–agarose-based hydrogel for four (CS-A-4), seven (CS-A-7), and ten (CS-A-10) days.

The CS-A hydrogel effect at the micro- and nanoscale on the remineralized layer’s microstructure is illustrated in Figure 5. The newly grown layer of CS-A-4 shows a prismatic rod with less organized nanorods in the central areas that is perpendicularly self-assembled on the enamel surface, as compared to the A-4 samples (Figure 3). Partial dissolution of the nanorods in the central area of the prismatic rod and the appearance of agglomerates in the interrod areas with some spherical formations were observed after seven (CS-A-7) and ten days (CS-A-10), respectively (Figure 5).

**Figure 6:**
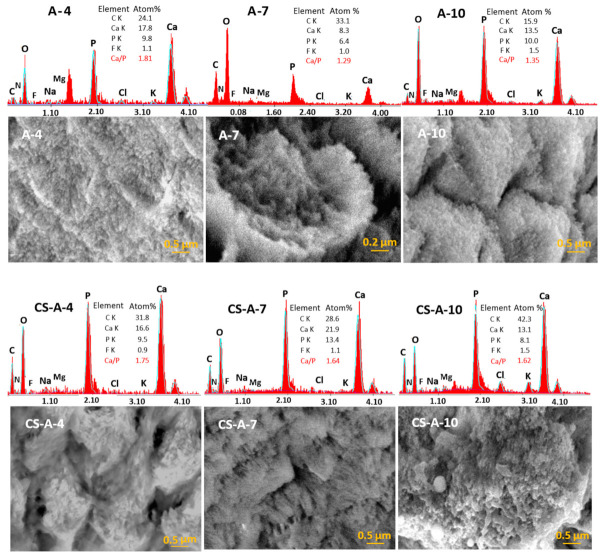
SEM-EDX data for remineralized samples under A-hydrogel (A-series) and CS-A-hydrogel (CS-A-series), respectively.

The elemental composition of the remineralized layers (Table 1, Figure 6) obtained by semi-quantitative SEM-EDX data [46] significantly differs in accordance with the hydrogel composition (with and without CS) and remineralization duration (4, 7, and 10 days). The value of the Ca/P ratio varies within 1.35–1.81 and 1.62–1.75 for the series remineralized under A-based hydrogel (series-A) and CS-A hydrogel (series-CS-A), respectively. While the values for the CS-A series are closer to the theoretical value of the carbonated hydroxyapatite of natural enamel (1.67), those for the series-A indicate the formation of calcium-deficient phosphates [47], whose composition worsens as the duration of remineralization increases. EDX spectra also give evidence of small amounts of fluoride, chloride, magnesium, sodium, and potassium ions. Fluorides are very likely present as fluorapatite.

**Figure 7:**
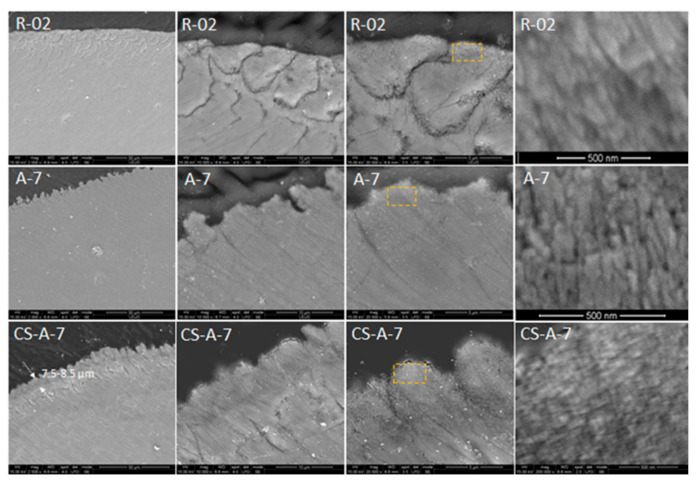
SEM image cross-sectional view of polished acid-etched natural reference enamel samples before (R-02) and after 7-day remineralization enamels in the presence of agarose (A-7) and chitosan–agarose (CS-A-7) hydrogels. (Yellow rectangle image is detailed on the right side.)

To estimate the thickness of the newly grown remineralization layers, the cross-sectional cut samples were embedded in epoxy resin and polished. Regardless of the possible implication of the sample preparation (cutting, embedding, and polishing), these contour areas certainly correspond to the interrod and organic sheath regions delimiting the enamel rod prisms, which at the external surface of the enamel layer have been more strongly affected by the chemical attack. The thickness of the grown layer of CS-A-7 was estimated at about 7–8 μm (Figure 7). Conversely, the seamless remineralized layer of A-7 prevents its thickness appraisal.

**Figure 8:**
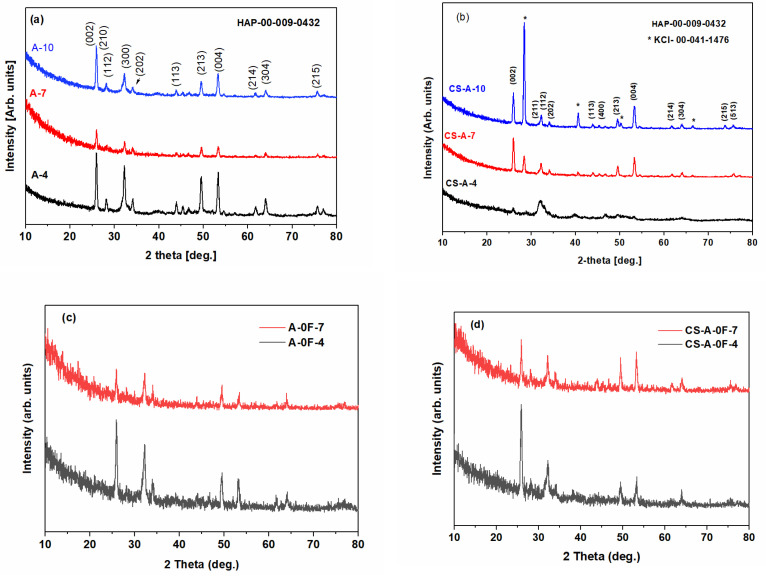
XRD patterns of remineralized samples: (**a**) A-(4/7/10), (**b**) CS-A-(4/7/10), (**c**) A-0F-(4/7), and (**d**) CS-A-(4/7) (Asterix stands for KCl).

The XRD patterns in Figure 8 of all remineralized layers with and without CS, after 4, 7, and 10 days of incubation show the characteristic diffraction lines of the hydroxyapatite (HAP) space group: P63/m (176) with the lattice parameters of a = b = 9.418 Å and c = 6.884 Å (JCPDS no. 09–0432). Thus, the sharp and intense (002) reflection peak in Figure 8 indicates that the grown HAP crystals are well crystallized and oriented along their c-axis, except for the CS-A-4 sample. The diffraction line around 2θ of 32° of a newly grown CS-A-4 layer is wider than that of CS-A-7/CS-A-10 layers, indicating low-crystalline HAP. Among the fluoride-free remineralized samples (Figure 8c,d and Table 1), the lowest HAP crystallinity was recorded for the CS-A-0F-4 sample. In addition, the CS-A-0F-(4/7) samples have lower HAP crystallinity than the ones remineralized in fluoride-containing saliva, CS-A-(4/7). In addition to the HAP crystalline phase, XRD patterns of CS-A-7 and CS-A-10 nanocomposites layers reveal the diffraction lines corresponding to KCl (JCPDS no. 041- 1476) (Figure 8a).

The values of the crystallinity degree, lattice parameters, and crystallite size of the HAP formed on the enamel surface are listed in Table 1. The average crystallite size of the grown HAP is smaller than that of native enamel for all studied samples except for the A-7 layer, which has an average crystallite size of 27 nm. The c values are very close to the reported values of the standard HAP phase. A decrease in the a-lattice parameter values is observed for all layers, suggesting an increase of carbonate content (carbonate ion enters the HAP structure in site B) [48,49]. The grown HAP crystals are well crystallized and oriented along their c-axis, except for the CS-A-4 sample, with an average crystallite size smaller than that of native enamel and an increase of carbonate ion site B of the HAP structure.

**Figure 9:**
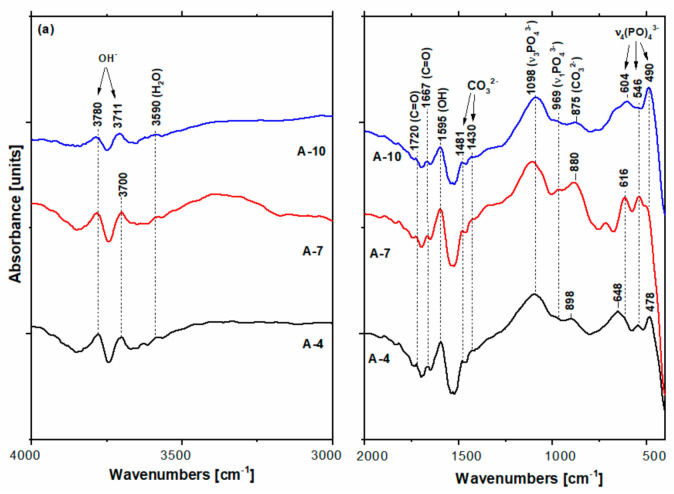

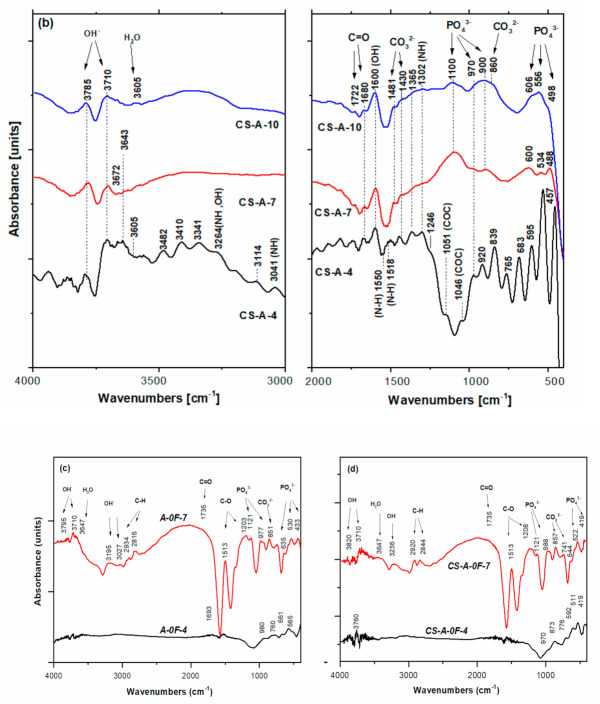
FTIR spectra of remineralized samples: (**a**) A-(4/7/10), (**b**) CS-A-(4/7/10), (**c**) A-0F-(4/7), and (**d**) CS-0F-(4/7).

The FTIR spectra of A and CS-A series are presented in Figure 9. In newly remineralized layers grown under the action of A-based hydrogel (Figure 9a) on the A-(4/7/10) samples, the FTIR bands of HO^−^, PO_4_^3−^, and CO_3_^2−^ groups in HAP were identified [49,50,51]. While the hydroxyl band positions of 3780 and 3590 cm^−1^ bands are insensitive to immersion, the vibrational modes of PO_4_^3−^ shifted from 616–478 cm^−1^ to 604 and 490 cm^−1^ after 10-day immersion. The intensities of the phosphate bands increase from sample A-4 to A-10, while the weakest 490 cm^−1^ and 546 cm^−1^ bands were recorded for A-7 and A-10, respectively. The concomitant presence of the 875, 1430, and 1481 cm^−1^ bands in the A-(4/7/10) spectra point out the formation of B-type apatite, namely carbonate substituted to phosphate ions [49,50,51]. The 873 cm^−1^ band of the CA-A-0F-4 is assignable to CO_3_^2−^. In addition, the shifted band position of the ν_2_(CO_3_^2−^) modes at ≥880 cm^−1^ for the A-4 and A-7 samples gives evidence of A-type HAP (some carbonate groups occupy hydroxyl sites) According to the band intensities, the richest carbonated HAP is present in the remineralized layer of A-7 enamel. Witzler [52] reported symmetric P-O stretching (ν_1_) at 962 cm^−1^, and O-P-O bending (ν_4_) at 602 and 561 cm^−1^ for composites with agarose. This is analogous with the grown layer of A-10. Although IR bands of carbonate containing HAP prevail in the A-series spectra in Figure 9a, the tiny bands at 1720 cm^−1^ and 1667 cm^−1^ attributable to C=O bonds are also noticeable [32].

Increasing of the HAP content of the newly grown layers on the CS-A samples as the immersion time increases is noticeable in Figure 9b. Wide bands within 3500–3000 cm^−1^ are due to the axial deformation of OH, NH, and intermolecular hydrogen bonds [27]. The bands located at 2920 and 2844 cm^−1^ originate from the symmetric and asymmetric C-H stretching modes of both agarose and chitosan [53]. The absorption bands at 3341 and 1302 cm^−1^ in Figure 9b are characteristic to the stretching of N-H in chitosan [27]. The shoulder located at 1046 cm^−1^ originates from bending modes of the C-O- in glucosidic bonds polysaccharide macromolecules of agarose [34]. This is dissimilar with the remineralized A-4 spectrum where the agarose hydrogel bands seemed to have nearly vanished. Carbonate bands [49] located at 1430 cm^−1^ and 840–860 cm^−1^ decrease in remineralized enamels of CS-A-7 and CS-A-10.

Carbonate groups in non-apatite environments were reported in the literature [50] to give an absorption band at about 866 cm^−1^ instead of 873 and 880 cm^−1^ for B- and A-type HAP. The three wide bands at 1100 cm^−1^, 900 cm^−1^, and 561 cm^−1^ corresponding to HAP [50] enhance in the remineralized layer of CS-A-10.

**Figure 10:**
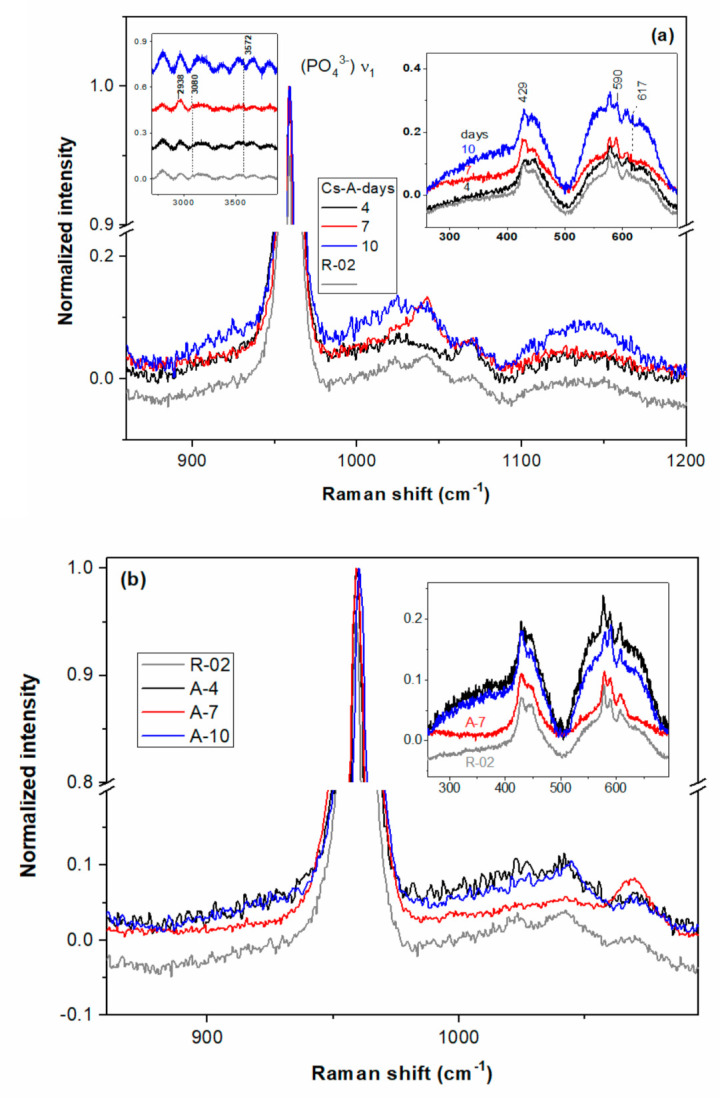

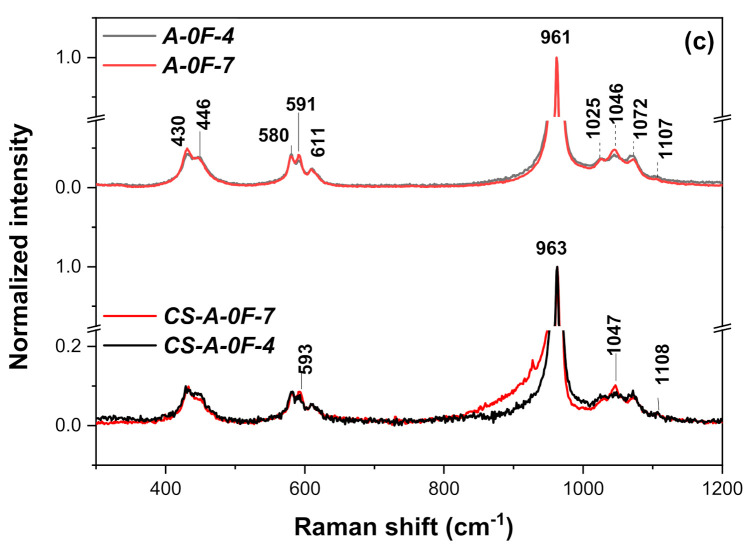
Raman spectra of etched R-02 and CS-A-(4/7/10) samples (**a**); A-(4/7/10) samples (514 nm laser); (**b**) (A/CS-A)-0F-(4/7) samples (325 nm excitation); (**c**) CS-4-A and R-02 spectra were down-shifted for convenience.

Raman spectroscopy is a useful technique to diagnose dental soft and hard structures in healthy, caried, and remineralized teeth as well as oral implants [52,54]. Raman spectral modifications among sound human enamel, eroded, and remineralized enamels arise mostly from the structure of the c-axis oriented enamel prisms [55,56]. Raman spectra of remineralized layers, A-(4/7/10) and CS-A-(4/7/10), are illustrated in Figure 10 in comparison with the ones of the acid-etched enamel of R-02 and fluoride-free remineralized (A/CS-A)-07-(4/7) samples, namely control group specimens. The left asymmetric ν_1_(PO_4_^3−^) band for the CS-A-0F-7 spectrum and presence of the 1108 cm^−1^ band (Figure 10c) give evidence of the HPO_4_^2−^ groups [51]. The P-OH vibration modes of the HPO_4_^2−^ groups was also depicted in the A-0F-7 spectrum (Figure 10c). The slight decreased intensity of the bands located at 446 and 1069 cm^−1^ and the intensity ratio of I_1070_/I_960_ (Table 1) were observed for the acid-etched R-02 sample in comparison with R-01, meaning a lower carbonate group content of the HAP crystals in etched enamel. Different peak intensities were recorded for the CS-A-(4/7/10) and A-(4/7/10) sets. More intense ν_1_(CO_3_^2−^) modes at about 1070 cm^−1^ in B-type HAP [56] were recorded for the CS-A-4 sample in contrast with the A-4 sample (see I1070/I960 in Table 1). In addition, triply degenerated ν_3_ (PO_4_^3−^) vibrations [56] peaking up at ≈1043 cm^−1^ are diminished for the CS-A-4 sample. As for bending modes at 590 (ν_4_) and 428 cm^−1^ (ν_2_) of PO_4_^3−^ [57], slight enhancement is noticeable for the A-4 sample (see Figure 10b).

Raman spectra of both CS-A-7 and A-7 resemble the one of R-02 except for more enhanced bands at 1043 cm^−1^ (CS-A-7) and 1069 cm^−1^ (A-7) due to the symmetric stretching vibrations of the PO_4_^3−^ and CO_3_^2−^ in the B-type HAP. Richer carbonate content triggers the decreasing of HAP crystallinity and its acidic dissolution [57]. Indeed, the full width at half maximum (FWHM) of the ν_1_(PO_4_^3−^) band increases in succession R-02 and CS-A-7 (≈9.95 cm^−1^) < A-0F-7 (≈11.05) > CS-A-0F-7 (≈11.4 cm^−1^) < A-7 (≈11.9 cm^−1^). The biggest I_1070_/I_960_ ratio among the 7-day remineralization treated samples was recorded for the A-0F-7 (0.0589) and CS-A-0F-7 (0.0574). Enhanced bands at 430, 590, and 1043 cm^−1^ for the CS-A-7 and CS-A-0F-7 spectra indicate richer c-axis oriented HAP content in comparison with carbonated HAP of the A-7 spectrum. An additional band at 2938 cm^−1^ of the CS-A-7 spectrum is due to ν(C-H) from chitosan (inset Figure 10a) [30,58]. Narrowing of the ν_1_(PO_4_^3−^) band after 10-day fluoride-containing saliva exposure is very likely to take place by partially acidic dissolution in a richer carbonate layer of A-7 [57].

### 3.2. Mechanical Characterization of Biomimetic Remineralized Enamel

The microhardness values of all air-dried remineralized samples in Table 1 are better than the one for acid-etched enamel. The 4-day remineralized sample (A-4) in the presence of agarose-based hydrogel recovered up to 93% (2,71 GPa) of the initial microhardness of the native enamel without chemical attack (R-01 in Table 1). The microhardness of the remineralized layers in this series decreases to ≈82% (2.39 GPa, A-7) and ≈76% (2.21 GPa, A-10) (Table 1) with increasing growth span at seven (A-7) and ten (A-10) days, respectively. Similar behavior can be observed for the microhardness of the new layers remineralized under CS-agarose hydrogel, noting that it decreases from 2.5 GPa (≈86%, CS-A-4) to 1.92 GPa (≈66%, CS-A-10) for the same immersion span.

## 4. Discussion

A chitosan–agarose polysaccharide-based hydrogel was developed to guide the biomimetic remineralization of acid etched dental enamels in artificial saliva. Different biomimetic grown layers were obtained under CS-A hydrogel in comparison with pristine agarose hydrogel. Formation of the enamel-like hierarchical structured layers under the two hydrogels mentioned above is also discussed in terms of maturation time in the following.

After 4-day treatment in artificial saliva, growing a new apatite crystallite in the preferentially c-axis perpendicular to the enamel surface is facilitated for the A-4 sample. The well-organized and uniform distribution of the characteristic nano- to microscale enamel-like structure of the remineralized synthetic layers of A-4 was depicted in SEM images in Figure 2. The axial section SEM micrographs of A-4 show that enamel rods and interrods, consisting of the same type of HAP nanocrystals, were epitaxially grown simultaneously with different orientations; this proves a successful biomimetic growth of artificial HAP on the acid-etched native enamel rods through specific and controllable individual epitaxial growth processes [45,59,60]. Less organized nanorods (SEM micrographs in Figure 5) with lower crystallinity (X-ray pattern in Figure 8b) might be coupled with more abundant c-misoriented HAP crystals in the remineralized layer on the CS-A-4 slice in comparison with A-4. An accentuated central prismatic rod growth, in contrast with rod sheaths height, caused the formation of exaggerated interrod spaces with porous appearance for CS-A-4. However, only a slight alteration of the fishscale structures occurs while the keyhole microstructures at larger scale were preserved (Figure 5). The higher carbonate content of the newly grown layer of CS-A-4 was recorded according to the higher intensity ratio of the carbonate and phosphate ions, I_1070_/I_960_ (Table 1), in the Raman spectrum. Since the tiny IR band at 1518 cm^−1^ (Figure 9b) is shifted in comparison with the one at 1509 cm^−1^ assignable to N-H vibrations in NH_3_^+^ groups of CS [34], the formation of a Schiff base (-C=N- groups) by a crosslinking reaction of chitosan with agarose was involved. The crosslinked A-CS system might be responsible for less organized rod growth of a depictable anchored HAP layer on to the CS-A-4 enamel revealed by SEM micrographs with respect to the A-4 sample. In addition, CS presence was depicted in the IR and Raman spectra of remineralized CS-A-4 in Figure 9b and Figure 10a. Intriguingly, a higher Ca/P value (1.81) than the stoichiometric crystalline carbonated HAP (1.67) was recorded for the A-4 sample, while the remineralized CS-A-4 layer encountered a ratio of 1.75 (Table 1). A slightly faster remineralization process under agarose action should be considered in the early stages of remineralization (see the Ca/P ratio in Table 1). A plausible explanation might consist of the faster ability of agarose to gel by forming interchain hydrogen bonds among OH groups and interaction with Ca^2+^ within an ionic crosslinked network [61]. Release of the excess Ca^2+^ cations from the ionic crosslinked agarose enables the formation of a calcium phosphate precursor for calcium-deficient HAP crystallites. At the same time, slower gelation and the weak affinity of chitosan for calcium [62,63] reduces the precipitation rate of HAP crystallites, which allows for a better control of their self-assembly and epitaxial growth on the demineralized enamel surface. Analogous to Cao’s findings [20], a lack of fluorides in the organic matrices used in enamel remineralization caused building of the needle-like structures on the (A/CS-A)-0F-4 samples. Fluoride addition highly influences the morphology of the biomimetic grown enamel-like structures.

Both remineralized (A/CS-A)-7 samples indicate a successful repairing of the enamel layer structure (Figure 7) by healing the subsurface lesions of the acid-etched enamel tissue followed by epitaxial growing of a newly synthetic HAP layer [45] without leaving any gap between the original enamel and resulting synthetic HAP layer. The enamel growing process is directed by the pre-existing crystalline nanorods of the substrate [43]. Despite the compact and homogenous structure of the remineralized A-7 and CS-A-7, they differ in distinguishing the newly grown layer. Thus, there is no clear distinction between the aspect of the native enamel and the newly grown layer for A-7, and therefore, its thickness cannot be estimated (Figure 7). The different microscopic aspect of the newly grown layer of A-7 can be explained based on modifications in chemical composition, density, and/or hardness (abrasion resistance). In contrast to an agarose repaired A-7 layer, the newly grown layer of CS-A-7 with a thickness of 7.5–8.5 μm shows the rounded top-end of the HAP rods (Figure 7). Another difference between the repaired enamels of A-7 and CS-A-7 consists in the Ca/P ratio of the newly grown enamel. Thus, a Ca/P ratio of 1.29 (Table 1) for the A-7 sample (without CS) suggests a calcium-deficient phosphate phase [47] as a result of dissolution of the nano calcium phosphate phases precipitated in the first step (A-4). Therefore, the fast grown HAP crystals on the remineralized A-4 underwent PO_4_^3−^ replacement by CO_3_^2−^ after 7-day immersion (A-7). The IR and Raman findings in Figure 9a and Figure 10b and I_1070_/I_960_ ratio in Table 1 for the remineralized layer of A-7 confirm its highest carbonate content and slightly less crystalline HAP (wider Raman band at about 960 cm^−1^). The SEM images in Figure 5 point out the structural modifications underwent by the CS-A slices, namely the formation of an initial new HAP phase onto CS-A-4, which subsequently dissolves partially and reprecipitates in the form of HAP nanorods (CS-A-7). The remineralized CS-A-7 has the closest Ca/P value to the sound enamel (Table 1). This is similar with the enamel remineralization under the combined effect of amelogenin-derived peptides and NaF when a greater mineral content of the repaired enamel than the one obtained with NaF alone was noticed [19]. In addition, ions carrying positive charged CS for tooth remineralization might enable hindrance of the biofilm formation due to streptococci colonization [63]. The growing of the c-axis HAP is just noticeable in the Raman spectrum of the CS-A-7 sample. The highest degree of HAP crystallinity (χc(002)), 0.47, 0.40, and 0.38, is observed for A-0F-7, A-7, and CS-A-7, respectively. However, the fluoride-free incubated sample has also a rich carbonate content (I_1070_/I_960_ ratio in Table 1), which is liable to dissolution. Despite the compact appearance of the needle-like enamel structures of the CS-A-0F-7 sample, we recorded the lowest crystallinity HAP. Swietlicka et al. [64] reported for the remineralized enamels under casein phosphopeptides and amorphous calcium phosphate a smaller A_1070_/A_960_ ratio, the area ratio derived from Raman spectra, than the sound enamel. This is the case of the CS-A-7 sample among the 7-day incubated samples.

The 10-day prolonged remineralization led to some structural modifications due to the more pronounced growth of an organic-rich interrod-sheath (A-10 in Figure 3). If the Ca/P ratio (see Table 1) of remineralized A-10 worsens very likely by the partial dissolution of its highly carbonated c-oriented HAP of A-7, the formation of spherical configurations and agglomerates in the interrod area is noticeable for newly grown layer of CS-A-10 (Figure 2). Unlike the A-10 sample (Figure 4), the microstructure of the remineralized CS-A-10 (Figure 5) is analogous to the remineralized HAP layers under EMD-CS hydrogel [30]. CS presence triggers the continuous improvement of the remineralized enamel composition with a small decrease in the Ca/P value (1.62) and at a degree of mineralization of 0.36 (χc) for the 10-day immersion process (Table 1). Thus, after a first slower step with the preferential growth of a new HAP phase in the rod core area (CS-A-4), a partial dissolution of the richer carbonate layer was observed with the highlighting of the interrod areas and precise contouring of the keyhole structures (CS-A-7), which was followed by a uniformly distributed re-precipitation of HAP nanocrystals and the formation of a KCl crystalline phase (CS-A-10). Lower hardness values for the hybrid CS-HAP layers (CS-A series) when compared with that of the A-series can be explained by the increased content of amorphous apatite phases, the presence of CS and KCl phases, as well as a growing of c-misoriented nanocrystals. Moreover, the organic component distribution significantly regulates the mechanical properties of enamel to better match its functional needs by restricting the movement of adjacent prismatic rods of HAP and the propagation of small cracks [65]. According to the data in Table 1, one can notice that the hardness modifications do not correlate straightforwardly with the variation of the Ca/P ratio, which decreases steeply from 1.81 to 1.29 for the A-series and decreases slightly from 1.75 to 1.62 in case of the CS-A series, but neither with the degree of crystallinity. During the early stage of remineralization, deep repair of the upper layer of the chemically attacked enamel, with the recovery to a great extent of the initial microhardness, takes place. Further treatment prolongation enables new HAP layers to be formed biomimetically but with lower microhardness than the sound enamel.

**Figure 11:**
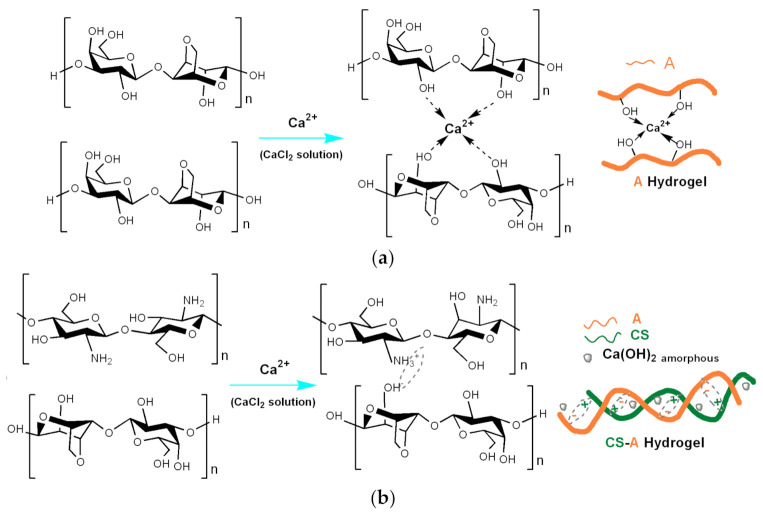
Formation of A-based hydrogel (**a**) and hybrid CS-A hydrogel (**b**) structure in the presence of Ca^2+^ ions.

The formation and growing of HAP on the acid-etched enamels in the A- and CS-A-sets of samples is rather different due to the functional groups (OH) of agarose and chitosan (NH_2_), which are respectively involved in the remineralization processes. Chitosan and agarose are marine polysaccharides derivate biopolymers, which aggregate and gel differently in ionic solutions [66,67,68,69]. This behavior could be attributed to the competition between OH and NH_2_ groups in forming Ca^2+^ clusters of different stability. In the case of agarose, the interaction that promotes the formation of stable Agarose-Ca-P clusters and their aggregation in oriented HAP crystals is between the hydroxyl group of agarose and calcium ions from hydrogel on the one hand and the surface of the etched enamel on the other [34]. Thus, the two sp^2^ “lone pair” of the oxygen atom within the hydroxyl group of the A-based hydrogel favors the acid–base Lewis interactions (Figure 11a). As a result, the intermolecular hydrogen bonding between the agarose chains and cross-linking interactions within Ca^2+^- agarose complex formation is competing, suppressing the association of agarose macromolecules in a helical configuration. The Ca^2+^- agarose crosslinking interactions along the polymer chain can explain the high microhardness recovery of the remineralized A-series (Table 1).

In recent years, it has been shown that the use of organic protein matrices for tooth enamel remineralization prevent the rapid precipitation of HAP crystallites, ensuring their controlled assembly into nanorods through specific interactions between Ca (II) ions on the face (001) of the HAP structure and NH_2_ groups of the protein molecules [67]. In the hybrid CS-A hydrogel at low acid pH (≈6.5), the CS polycations by its NH_3_^+^ groups trigger the formation of helical configurations through electrostatic interactions with the poly-anionic agarose chains (Figure 11b) [69]. Moreover, superficial precipitations of HAP on deteriorated human enamel are highly pH-dependent [46]. These CS-A interactions reduce the crosslinking through Ca^2+^ ions and favor their precipitation with the formation of hydrogels with complex structures (Figure 11b). We suppose that the small amorphous precipitates from the hybrid hydrogel may represent small deposits from which calcium ions are released in a controlled manner during the remineralization process, which would explain the presence of less crystallized HAP in the early remineralized CS-A-4 layer (Figure 4). In the present case, the matrix represented by the CS-A polysaccharides hydrogel has demonstrated the hydrogen bonding interactions between the chitosan and agarose macromolecules with the formation of a fibrous structure [34] in which, most likely, the competition between the interactions of OH of agarose ions and NH_2_ groups of chitosan with ions Ca^2+^ leads to a complex hybrid chemical structure, which requires a more detailed investigation (next study).

## 5. Conclusions

Early-stage eroded dental enamel regulated by a novel hydrogel matrix of agarose and chitosan was carried out in vitro biomineralization. All the grown hydroxyapatite layers under A and CS-A hydrogels in fluoride-containing artificial saliva show enhanced microhardness values in comparison to the acid-etched enamel. Despite the better microhardness recovery of the agarose remineralized enamel layers in comparison with CS-A remineralized ones, its calcium-deficient HAP points out a liability toward further chemical attack. Thus, the main difference between the new grown HAP layers for 7 days in the presence of the A-based hydrogel and CS-A hybrid hydrogel consists in the accentuated growth in the central area of the prismatic rod (consisting of nanorods with larger diameter) in the first case (A-7) and an accentuated increase of the interrods that form the specific “key-hole” enamel structure with smaller nanorod diameter in the central area in the presence of chitosan (CS-A-7). Further prolongation of the immersion time under the CS-A system causes the formation of non-apatite carbonate and chitosan traces in the remineralized layer, which imply defective splicing of the c-oriented HAP rods with mechanical implications.

Our results not only confirmed that natural polysaccharide CS biopolymers provided an array to self-assemble HAP nano-building units but also acted as moderating agent in HAP nanorods formation and reducing carbonation. Therefore, CS can act as a bioactive macromolecule in the biomimetic reconstruction of an enamel hierarchical structure and CS-A hydrogel can mimic the protein matrix for cost-effective multifunctional layers in enamel treatment.

## Figures and Tables

**Table 1:** Structural and compositional parameters of the remineralized enamel samples and the native (R-01) and acid-etched (R-02) enamel samples.

Sample	2θ (deg) (002)	FWHM (deg)	Ca/P (EDX Data) (at %/wt %)	I_1070_/I_960_ (Raman Data)	Crystallite Size (002) (nm)	χ_c_ (002)	Lattice Parameters ^#^		Microhardness (GPa)
							A = b(Å)	c(Å)	
**Remineralized Samples in the Presence of Chitosan (CS–Agarose Hydrogel)**									
CS-A-4	25.844	0.3413	1.75/2.26	0.0593	24.9(0.3)	0.34	9.408(1)	6.891(1)	2.50
CS-A-7	25.968	0.3319	1.64/2.12	0.0474	25.6 (0.4)	0.38	9.406(1)	6.891(1)	2.26
CS-A-10	25.958	0.3359	1.62/2.07	0.0457	25.3 (0.5)	0.36	9.439(2)	6.883(1)	1.92
**Remineralized Samples without Chitosan (Agarose Hydrogel)**									
A-4	25.947	0.3487	1.81/2.31	0.0442	24.4 (0.3)	0.33	9.407(1)	6.897(1)	2.71
A-7	26.02	0.3222	1.29/1.61	0.0699	27.6 (1.6)	0.40	9.400(3)	6.893(2)	2.39
A-10	25.958	0.3455	1.35/1.73	0.0424	24.6 (0.6)	0.34	9.409(1)	6.895(1)	2.21
**Remineralized Samples in Fluoride-Free Artificial Saliva under Agarose and Chitosan Hydrogels**									
A-0F-4	25.9439	0.3447		0.0569	24.7 (1.7)	0.33	9.270(2)	6.910(16)	
A-0F-7	25.8797	0.3058		0.0589	27.8 (3.8)	0.47	9.30 (3)	6.884 (3)	
CS-A-0F-4	25.8851	0.3661		0.0562	23.2 (1.3)	0.28	9.23(18)	6.912(5)	
CS-A-0F-7	25.9634	0.3618		0.0574	23.5 9 (2)	0.29	9.365(17)	6.905(9)	
**Reference Samples—** **Native Enamel (as Selected or Acid Etched)**									
R-01	-	0.3128		0.0463	27.2(0.5)	0.46			2.92
R-02	25.96	0.3755		0.0425	22.7(1.0)	0.28			1.68

# Hydroxyapatite—crystal system hexagonal; space group P63/m(176)-00-09-0432.

## Data Availability

Not applicable.
